# Investigation of the Real-Time Release of Doxycycline from PLA-Based Nanofibers

**DOI:** 10.3390/jfb14060331

**Published:** 2023-06-20

**Authors:** Noémi-Izabella Farkas, Laura Marincaș, Lucian Barbu-Tudoran, Réka Barabás, Graziella Liana Turdean

**Affiliations:** 1 Department of Chemical Engineering, Faculty of Chemistry and Chemical Engineering, Babeș-Bolyai University, 11 Arany János Street, 400028 Cluj-Napoca, Romania; noemi.farkas@ubbcluj.ro; 2 Department of Chemistry, Faculty of Chemistry and Chemical Engineering, Babeș-Bolyai University, 11 Arany János Street, 400028 Cluj-Napoca, Romania; laura.marincas@ubbcluj.ro; 3 Department of Molecular Biology and Biotechnology, Faculty of Biology and Geology, Babeș-Bolyai University, 1 Mihail Kogălniceanu Street, 400084 Cluj-Napoca, Romania; lucian.barbu@ubbcluj.ro; 4 National Institute for Research and Development of Isotopic and Molecular Technologies, 67-103 Donath Street, 400293 Cluj-Napoca, Romania; 5 Department of Chemistry and Chemical Engineering of Hungarian Line of Study, Faculty of Chemistry and Chemical Engineering, Babeș-Bolyai University, 11 Arany János Street, 400028 Cluj-Napoca, Romania

**Keywords:** nanofibers, doxycycline, drug release, real-time monitoring, differential pulse voltammetry

## Abstract

Electrospun mats of PLA and PLA/Hap nanofibers produced by electrospinning were loaded with doxycycline (Doxy) through physical adsorption from a solution with initial concentrations of 3 g/L, 7 g/L, and 12 g/L, respectively. The morphological characterization of the produced material was performed using scanning electron microscopy (SEM). The release profiles of Doxy were studied in situ using the differential pulse voltammetry (DPV) electrochemical method on a glassy carbon electrode (GCE) and validated through UV-VIS spectrophotometric measurements. The DPV method has been shown to be a simple, rapid, and advantageous analytical technique for real-time measurements, allowing accurate kinetics to be established. The kinetics of the release profiles were compared using model-dependent and model-independent analyses. The diffusion-controlled mechanism of Doxy release from both types of fibers was confirmed by a good fit to the Korsmeyer–Peppas model.

## 1. Introduction

Polymer nanofibers produced through the electrospinning technique are often used as drug delivery systems (DDS) because of their strong affinity for active substances [1]. The homogenous and continuous nanofibers prepared by electrospinning have advantageous physicochemical properties in terms of drug loading [2]. Nanofibers made from synthetic and biodegradable aliphatic polyesters (e.g., poly (glycolic acid) (PGA) [3], poly (lactic acid) (PLA) [4], poly (caprolactone) (PCL) [5] and polydioxanone (PDO) [6]) can be used as DDS because of their biocompatibility, large specific surface area, controllable and modifiable surface, and complex porosity.

Drug release can be influenced by the preparation method of drug-loaded nanofibers, with the most commonly used methods being chemical bonding, core/sheath, or encapsulation [7]. The controlled release of the active substance from these systems is influenced by the compatibility between the solvent and drug, as well as the polymer and drug [8]. As a result, the rate of release from the nanofibers is positively correlated with the surface area of the fibers [9]. In addition to the various drug encapsulation methods, the active substance can also be loaded during the so-called post-electrospinning process [10]. Among the methods employed for the post-electrospinning process, the simplest one-step method is the physical adsorption of the drug. In this case, no chemical bond is formed between the polymer and the active substance, and the drug release is determined by secondary interactions and drug recrystallization during adsorption. However, the final release of the active substance is influenced by the properties of both the polymer and the drug (hydrophobic or hydrophilic) [10,11]. Consequently, release from polymer nanofibers can be characterized as either a burst release or a slow, regular drug-release profile.

Research studies on PLA nanofibers often used as DDS have shown that a larger fiber diameter has a positive effect on the constant release of the active substance [12]. In a one-day in vitro study, a significant burst release of tetracycline hydrochloride from PLA nanofibers was observed. This rapid drug release was explained by weak physical interactions between the model drug and PLA [2]. Long-term sustained release was achieved using composite PLA nanofibers [13]. Different bioceramic nanomaterials can also be used as additives, especially to improve the biological properties of PLA nanofibers. H. R. Bakhsheshi-Rad et al. used the electrospinning method to prepare PLA nanofibers containing akermanite and doxycycline on magnesium alloy plates for bone tissue engineering and subsequently characterized them. Their studies initially showed rapid release due to the weak hydrogen bonds between Doxy and PLA, followed by a sustained release phase [14]. In recent years, hydroxyapatite composite nanofibers (e.g., PLA/Hap) have been studied for similar purposes [15,16,17].

In vitro examination of active drug release is usually conducted using conventional methods such as high-performance liquid chromatography (HPLC) or ultraviolet-visible (UV-VIS) spectroscopy. Although these methods are precise, they have significant drawbacks due to the limitations in continuous measurement [18,19]. In addition, the main disadvantages of HPLC are its high operating costs and long detection period [20]. Sampling often causes problems because of the need to filter or centrifuge the sample before measurement [21]. In addition, the simultaneous examination of two different active substances in the same solution is impossible using UV-VIS [9].

Because most active substances contain electroactive groups, electrochemical investigation methods, particularly voltammetric methods, are becoming more and more common. Voltammetric methods, using either modified or unmodified working electrodes, have also been employed for antibiotic detection [22,23] and drug-release studies [24,25,26]. The main advantage of voltammetric methods is real-time monitoring so the drug release can be determined directly in situ. In the case of these measurements, the interference caused by the presence of the undissolved drug in the release media is avoided [24]. Differential-pulse voltammetry (DPV) and square-wave voltammetry (SWV) are commonly used methods in this field of research. Compared to other instrumental techniques, they are more cost-effective, with high sensitivity and precision, so the measured dissolution profiles can show realistic kinetics [20,21]. The in situ release of paracetamol from symmetric and asymmetric sugarcane bagasse membranes [27], as well as from thin chitosan and agarose films [28], was successfully investigated using the SWV method. The applicability of the method was studied for examining the release of piroxicam [29], doxorubicin [18], naproxen [21], selegiline [30], etc. DPV is also a remarkable alternative method for the qualitative and quantitative investigation of drug dissolution [23,31,32]. The oxidation of the analyte and the corresponding current production are caused by voltage pulses of increasing amplitudes. The oxidation peak that appears is characteristic of the analyzed analyte, and the intensity of the peak current is linearly proportional to the concentration (within a certain range) [19].

Doxycycline (Doxy) is a commonly used antibiotic belonging to the tetracycline group [33]. The oxidation process of the voltammetric behavior of Doxy has been described in several studies. In general, two oxidation peaks are observed, the first being due to the dimethylamino group in position 4, and the second to the phenolic group in position 10 [34,35]. Studies have shown that increasing the pH decreases the peak current intensity, which is characteristic of the phenolic group in the 10-position of tetracyclines [36,37]. However, most of these studies focus on the detection of Doxy in different foods [38] or pharmaceuticals [39].

The present work aimed to investigate the Doxy release process from PLA and PLA/Hap nanofibers in situ using the DPV method. Although the release from similar PLA and PLA/Hap nanofibers has already been investigated in our previous study using the UV-VIS spectrophotometric method with continuous sampling [40], the investigation of Doxy release from DDS using the DPV method is less common. However, this method offers real-time monitoring of the release of the active substance and allows for more accurate measurements of release kinetics. The method was validated using the UV-VIS spectrophotometric method. Model-dependent and model-independent approximations were used to study and compare the release kinetics.

## 2. Materials and Methods

The required reactants for Hap synthesis, i.e., Ca(NO_3_)_2_·4H_2_O (purity ≥ 99%) and (NH_4_)_2_HPO_4_ (purity ≥ 98%), were obtained from Carl Roth GmbH (Germany). PLA and PLA/Hap nanofibers were prepared from PLA granules with a 3 mm nominal granule size, distributed by Sigma Aldrich. Chloroform (99.0–99.4% (GC) was obtained from Honeywell, dichloromethane (purity ≥ 99.5%) was purchased from VWR Chemicals, and doxycycline hyclate (purity ≥ 93.5%) was purchased from Sigma Aldrich. The salts required for the phosphate buffer solution (PBS) (8 g·L^−1^ NaCl, 0.28 g·L^−1^ KCl, 1.448 g·L^−1^ Na_2_HPO_4_, 0.248 g·L^−1^ KH_2_PO_4_) were purchased from Sigma Aldrich and “Reactivul” București. The materials used for all experiments were of analytical purity and were used without subsequent purification processes.

### 2.1. Preparation of Hap

Hap was prepared according to our previously reported method [41], but a brief description of the synthesis is presented below. The (NH_4_)_2_HPO_4_ aqueous solution with a pH of 11 was added to the Ca(NO_3_)_2_ × 4 H_2_O solution with the same pH using a peristaltic pump under constant stirring. The mixture was stirred for 24 h and then filtered and washed with distilled water. For the subsequent steps, i.e., the production of PLA/Hap nanofibers, Hap was used as a precipitate.

### 2.2. Preparation of PLA and PLA/Hap Nanofiers

PLA and PLA/Hap nanofibers were produced through the electrospinning technique using a Fluidnatek^®^ LE-50 benchtop laboratory machine (Bioinicia S.L., Valencia, Spain). An 8 wt% PLA solution was prepared using a mixture of DCM/CHL in a ratio of 6:4 (*v/v* %). For the preparation of PLA/Hap nanofibers, the Hap precipitate was added to the PLA solution, and the PLA/Hap ratio was 0.8. To achieve adequate homogeneity, the PLA/Hap mixture was stirred for 24 h on a magnetic stirrer. During the electrospinning process, both polymer mixtures were spun at a flow rate of 800 µL/h, with a needle tip-collector distance of 15 cm and a voltage of 27 kV. These parameters were chosen based on our previous experiments [40]. To easily remove the resulting nanofiber mats, a baking paper sheet was used as a collector.

### 2.3. Nanofiber Characterization Methods

The morphologies of the PLA and PLA/Hap nanofibers were examined through scanning electron microscopy (SEM) using a CFEG SEM Hitachi SU8230 microscope (Hitachi-shi, Japan). ImageJ software was used to measure the diameter distribution of the nanofibers.

### 2.4. Adsorption of Doxy on Nanofibers

Doxy was loaded onto the PLA and PLA/Hap electrospun mats using the physical adsorption method with different initial concentrations of Doxy (i.e., 3, 7, and 12 g/L in double-distilled water) to obtain PLA_Doxy_x and PLA/Hap_Doxy_x composite matrices (where x is the initial concentration of Doxy solution). During the adsorption process, 50 mg of nanofiber was shaken with 5 mL of the Doxy-containing aqueous solution of the corresponding concentration using Biosan Bio RS-24 mini-rotator equipment. The drug’s adsorption was measured after 24 h in the liquid phase using a Jasco V-650 UV-VIS double-beam spectrophotometer (Tokyo, Japan) at 274 nm. The measurement was repeated three times, and the average adsorption capacity of the obtained fibers was presented and discussed. The Doxy-containing nanofibers were dried until a constant mass was achieved.

### 2.5. Voltammetric and Spectrophotometric Investigation of Drug Release

Cyclic voltammetry (CV) and differential-pulse voltammetry (DPV) were performed using a computer-controlled potentiostat (AutoLab, type PGSTAT302N, EcoChemie, Utrecht, The Netherlands) operated by GPES 4.7 software. Measurements were carried out using a conventional three-electrode system comprising a platinum wire auxiliary electrode, a Ag/AgCl, KCl_sat_, reference electrode and a glassy carbon electrode (GCE). The influence of the pulse amplitude (i.e., 10–100 mV) and step potential (i.e., 1–20 mV) on the peak current intensity led to the optimization of the experimental operating voltammetric parameters.

For the voltammetric determination of Doxy release, the electrochemical cell contained the dried Doxy-loaded electrospun mats (typical amount: 50–60 mg) and 20 mL of phosphate buffer solution (PBS, pH = 7). Doxy release was measured for each sample at predetermined intervals over 12 h (i.e., 15, 30, 45, 60, 75, 90, 105, 120, 135, 150, 165, 180, 240, 300, 360, 420, 480, 720 min). The measurements were performed in the potential range of +0.3 and +1.3 V vs. Ag/AgCl, KCl_sat_, using the optimal parameters: a 50 mV pulse amplitude and a 5 mV step potential, with a 1.5 V conditioning potential for 20 s. These operating parameters were used for both the calibration curve and measuring the release of the active substance.

In order to validate the DPV method, the drug-release study was also performed spectrophotometrically at a wavelength of 274 nm. Similar to the voltammetry measurements, the fibers containing varying amounts of Doxy were immersed in 20 mL of PBS, with their mass approximately equal to the mass used in the DPV measurements. Sampling was performed at the same time intervals, where the 2 mL sample used for the measurements was replaced with fresh PBS to maintain a constant medium volume. All DPV and UV-VIS measurements were repeated 3 times.

### 2.6. Drug-Release Study

Drug-release kinetics were investigated from two approaches: (i) the suitability of the DPV method for measuring drug release was investigated using the model-independent method, and (ii) the parameters affecting dissolution were discussed using a model-dependent approach (mathematical models describing the release kinetics). The model-independent method recommended by the FDA (Food and Drug Administration) is based on the calculation of similarity (*f*_2_) and difference (*f*_1_) factors. In general, it is recommended to calculate both factors to establish a certain equivalence between two different release profiles [42]. The prerequisite for this is that the value of *f*_1_ < 15 and the value of *f*_2_ > 50. In the present study, the release profile measured by DPV was compared with the UV-VIS measurement data series. The following formulas were used for calculations:

(1) $$f_{1}=\frac{\sum_{t=1}^{n}\left(R_{t}-T_{t}\right)}{\sum_{t=1}^{n}R_{t}}\times 100$$

(2) $$f_{2}=50\times log\left[{\left\{1+\frac{\sum_{t=1}^{n}{\left(R_{t}-T_{t}\right)}^{2}}{n}\right\}}^{-0.5}\right]\times 100$$

where *n* is the number of measured points, *R_t_* is the dissolution value of the reference (UV-VIS profile) at time *t*, and *T_t_* is the dissolution value of the test (DPV profile) at time *t*.

A linear regression analysis was performed during the model-dependent analysis. The determination coefficient (R^2^) was compared for the zero-order kinetic, first-order kinetic, Higuchi, Hixson–Crowell, and Korsmeyer–Peppas models, respectively.

## 3. Results and Discussion

### 3.1. Morphological Investigation by SEM

**Figure 1:**
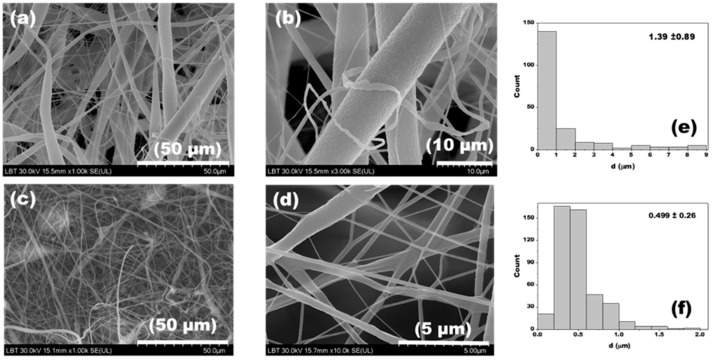
SEM images of PLA (**a**,**b**) and PLA/Hap (**c**,**d**) nanofibers and their corresponding fiber-diameter distributions (**e**,**f**).

Figure 1a–d show SEM images of the pure PLA and PLA/Hap electrospun mats at two different resolutions. In both cases, randomly oriented nanofibers formed a network-like structure with a continuous and relatively smooth surface. At higher resolution, the fibers exhibited a slightly rough surface morphology, which is also suitable for biomedical use [43]. The distributions of nanofiber diameters were irregular, with the average diameter ranging from 1.39 ± 0.89 µm for PLA nanofibers to 0.499 ± 0.26 µm for PLA/Hap. As observed, the addition of Hap precipitate to the PLA solution led to a significant decrease in the average fiber diameter. The reduction in diameter of composite nanofibers formed with organic/inorganic components has been previously discussed by D. Chuan et al. [44], who stated that the addition of Hap to the polymer solution changed its conductivity or viscosity. Consequently, as the values of these parameters increased, the fiber diameter decreased [45,46]. On the other hand, a narrow diameter distribution of PLA/Hap nanofibers compared to pure PLA nanofibers was observed.

In the case of PLA electrospun mats, it was noticeable that the diameters of the individual nanofibers were often not the same. In the image with a resolution of 50 µm (Figure 1a,c), fibers with significantly varying thicknesses (5–9 µm) were observed. This phenomenon was explained [47] by the insufficient evaporation of the solvent. A possible reason for this behavior was the small distance between the tip of the needle and the collector in the electrospinning process.

### 3.2. Adsorption Study

**Figure 2:**
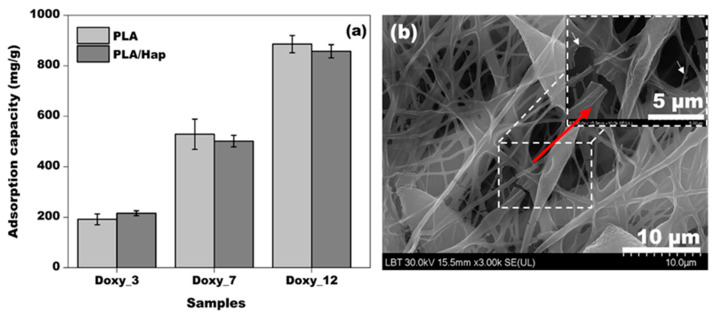
Adsorption capacity of PLA and PLA/Hap nanofibers with different concentrations of Doxy (**a**), and SEM image of the PLA_Hap_Doxy_12 nanofiber (**b**). Error bars represent measurements repeated 3 times.

Figure 2a shows the average adsorption capacity of Doxy on PLA and PLA/Hap electrospun mats. A similar adsorption capacity was observed for both nanofiber systems, with an average adsorption efficiency of 70–80% and a small standard deviation (5–15%) based on three measurements. This high adsorption efficiency can be explained by the high specific surface/volume ratio characteristic of nanofibers [48] and the spatial orientation and network structure of the fibers (See Figure 1a). Additionally, it was observed that the adsorption capacity of the nanofibers increased with the initial concentration of Doxy, indicating a stronger driving force for the adsorption process [49,50]. The continuous increase in adsorption capacity due to the initial Doxy concentration indicates that the saturation of active sites on the fibers was not reached during the process. The amount of adsorbed Doxy on the fibers was 10.7 mg for the 3 g/L initial Doxy solution and 55.3 mg for the 12 g/L initial Doxy solution. The chemical composition of the nanofibers did not significantly affect the final amount of adsorbed Doxy. Depending on the initial concentration of Doxy, the difference in adsorption capacity between the PLA/Hap and PLA fibers was negligible. This similarity in adsorption capacity suggests that the added Hap did not block the adsorption of active sites.

Figure 2b shows an SEM image of the PLA/Hap_Doxy_12 sample. It can be seen that the shaking during adsorption caused slight fragmentation in the electrospun mats. Thus, the adsorbed Doxy was observed as agglomerates on the surface of the fibers, which are indicated by white arrows in the inset image.

### 3.3. Electrochemical Measurements

#### 3.3.1. Cyclic Voltammetry

**Figure 3:**
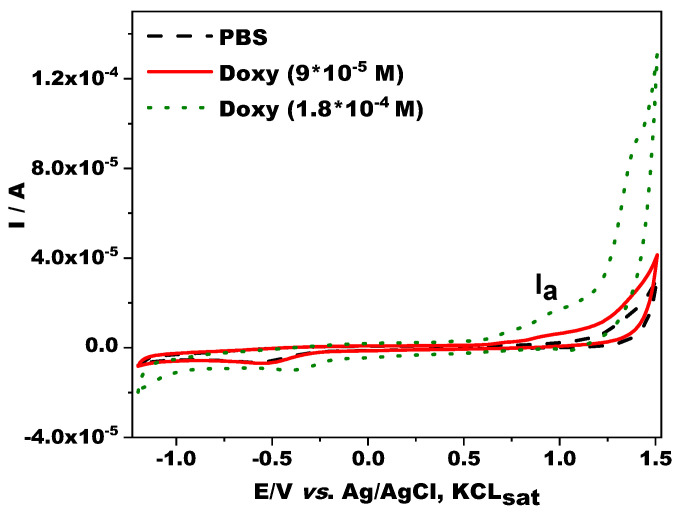
Cyclic voltammograms in the absence and presence of Doxy on the GC electrode. Experimental conditions: electrolyte, 0.1 M PBS (pH 7); scan rate, 50 mV/s; starting potential, −1.2 V vs. Ag/AgCl, KCl_sat_.

Cyclic voltammetry was performed to study the redox behavior of Doxy on a GCE electrode. Figure 3 shows the cyclic voltammograms in the presence and absence of Doxy in PBS solution (pH 7). As expected for typical tetracyclines [34], an anodic peak (I_a_) attributed to the oxidation of Doxy appeared at a potential of +0.935 V vs. Ag/AgCl, KCl_sat_, irrespective of the Doxy concentration, covering the concentration range used for the calibration. Although the investigation of drug release with the CV method has been reported in the literature [51], the DPV method is a more sensitive analytical technique so it was used for the examination of the drug-release study.

#### 3.3.2. Optimization of DPV Operating Parameters

The influence of the input signal parameters (i.e., step potential and pulse amplitude) used in the DPV investigation method was studied in order to determine the values that yielded the maximum intensity of the Doxy oxidation peak.

**Figure 4:**
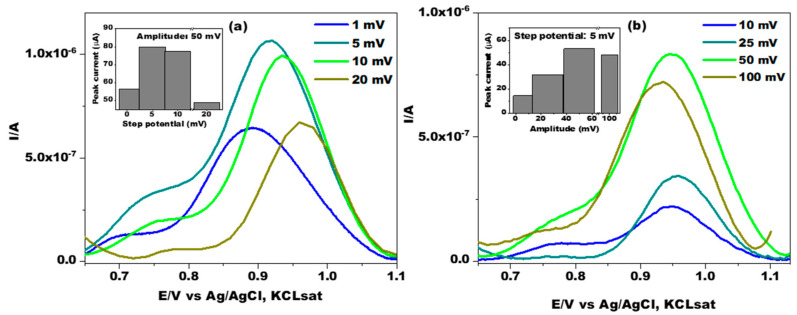
Influence of the step potential (**a**) and pulse amplitude (**b**) on DP voltammograms recorded on the GCE in the presence of 1.4 × 10^−4^ M Doxy, and the corresponding anodic peak current dependencies (inset). Experimental conditions: electrolyte, 0.1 M PBS (pH 7); pulse amplitude, 50 mV; step potential, 5 mV; starting potential, 0.7 V vs. Ag/AgCl, KCl_sat_.

In the first step, the influence of the step potential (1, 5, 10, and 20 mV) on the Doxy oxidation peak current was investigated. In order to choose the best potential step value, DP voltammograms were recorded by applying a pulse amplitude of 50 mV. It is clear from Figure 4a that the peak current reached its maximum value at 5 mV. Similarly, the effect of varying the pulse amplitude (e.g., 10, 25, 50, and 100 mV) on the peak current was also studied at a step potential of 5 mV. From Figure 4b, it can be seen that the Doxy peak current increased linearly with the increase in the pulse amplitude and then reached its maximum value at 50 mV. Consequently, to obtain relatively high and narrow peaks in subsequent experiments, values of 50 mV and 5 mV were chosen for the pulse amplitude and step potential, respectively.

#### 3.3.3. Monitoring of Doxy Release by Differential Pulse Voltammetry

**Figure 5:**
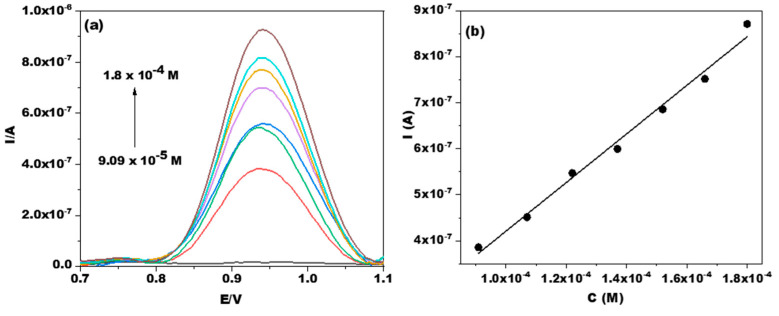
DPV voltammograms on the GCE for different concentrations of Doxy (**a**) and the corresponding calibration curve (**b**). Experimental conditions: electrolyte, 0.1 M PBS (pH 7); pulse amplitude, 50 mV; step potential, 5 mV; starting potential, +0.7 V vs. Ag/AgCl, KCl_sat_.

The real-time release of Doxy was investigated using the DPV method with the optimal operating parameters (Figure 5a). The calibration curve was repeated three times, obtaining the following mean equation: I_p_/A = (−10 × 10^−6^ ± 3.33 × 10^−8^) + (0.0053 ± 2.39 × 10^−4^) × [Doxy]/M), with a coefficient of determination R^2^ = 0.9898, based on 7 data points. In addition, the linear domain of the electrode function was found in the concentration range of 0.9 × 10^−4^–1.8 × 10^−4^ M Doxy (Figure 5b). Subsequently, the obtained calibration curve equation was used to calculate the amount of Doxy released from the PLA and PLA/Hap nanofiber matrices. Furthermore, the limit of detection (LOD) and limit of quantification (LOQ) were calculated according to the equations LOD = 3 × S_da_/m and LOQ = 10 × S_da_/m, where “S_da_” is the standard deviation of the intercept of the calibration curve and “m” is the slope of the calibration curve [52]. The calculated LOD and LOQ values were 1.9 × 10^−5^ M and 6.32 × 10^−5^ M Doxy, respectively. It can be concluded that the analytical parameters estimated for the determination of Doxy by DPV have satisfactory values, and the DPV investigation method is sufficiently suitable for studying the in situ release of Doxy from PLA and PLA/Hap electrospun mats.

**Figure 6:**
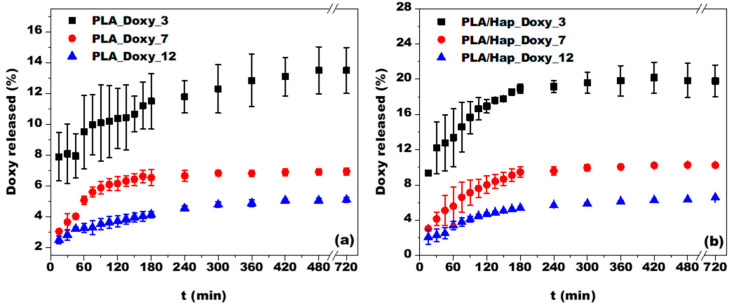
Doxy release investigated using the DPV method on PLA (**a**) and PLA/Hap (**b**) electrospun mats. Experimental conditions: electrolyte, 0.1 M PBS (pH 7); pulse amplitude, 50 mV; step potential, 5 mV; starting potential, +0.7 V vs. Ag/AgCl, KCl_sat_; mean and standard deviation for 3 successive measurements.

The average release profile of Doxy from PLA and PLA/Hap electrospun mats is shown in Figure 6a,b. As shown, the release of Doxy from both studied systems showed a very similar trend, with an increase in the amount of dissolved Doxy over time. Throughout the 12 h experiment, the dissolution profiles showed sustained release, with no case exceeding 20% of the total Doxy amount released. The sustained drug release from PLA-based nanofibers is consistent with findings reported in the literature [53,54]. Previous research has proven the possibility of using this type of fiber for the prolonged release of drugs or biomolecules [55,56]. The investigated systems may have physiological significance due to the controlled Doxy release [55].

In the first 3 h, a large amount of drug release was observed in all systems, followed by a slow release, which was the result of the low dissolution rate. The initial burst release may have been due to the recrystallization of Doxy [10], its low molecular weight, and its good affinity for PBS [57]. This behavior may have also been influenced by the weak secondary bonds between Doxy and the electrospun mats, leading to the weakly bound Doxy molecules being released during the first 3 h of the experiment. Moreover, the literature mentions that drug release can be enhanced by drug–drug interactions [58], which was confirmed in our case by the formation of Doxy aggregates (Figure 2b). From Figure 6a,b, it can be observed that in the system with the smallest Doxy content, the release of the drug was greater over time (13.5% in the case of PLA_Doxy_3 and 19.8% in the case of PLA/Hap_Doxy_3). These systems showed the least reproducible behavior since the standard deviation (in the amount of Doxy released) was 2–6.5% for PLA_Doxy_3 and 0.2–3.3% for PLA/Hap_Doxy_3.

The samples prepared using solutions with initial concentrations of 7 g/L and 12 g/L of Doxy showed the best reproducibility and the lowest standard deviation between individual measurements. For these systems, the dissolution trend was quite similar, depending on the Doxy content. After 3 h of examination, the systems showed a steady-state behavior, characterized by a sustained, constant release of drugs from the nanofibrous matrix, probably due to the large fiber diameter (>500 nm) [12]. The presence of Hap in the Doxy encapsulation matrix led to a higher amount of Doxy being released than from the simple PLA fiber matrix. For example, after 12 h, the Doxy release was 6.9% from the PLA_Doxy_7 sample and 10.2% from PLA/Hap_Doxy_7 sample. Similarly, in the case of the PLA_Doxy_12 sample, a release of 5.1% was measured, whereas in the case of PLA/Hap_Doxy_12, it was 6.6%. Even in these systems, most of the released Doxy occurred in the first 3 h.

### 3.4. Correlation between DPV and UV-VIS Drug-Release Studies

**Figure 7:**
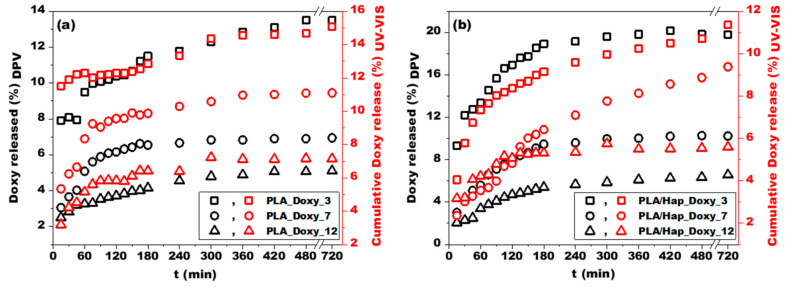
Doxy release profiles obtained through DPV (black scatters) and UV-VIS (red scatters) for PLA (**a**) and PLA/Hap-based samples (**b**) loaded with Doxy. Experimental conditions: for DPV measurements, see Figure 6; for UV-VIS measurements, wavelength = 274 nm.

In order to validate the Doxy release profiles obtained through the DPV electrochemical method, UV-VIS spectrophotometry measurements were performed. The average released Doxy values (expressed in %) measured by DPV and UV-VIS are illustrated in Figure 7a,b. The spectrophotometric measurements are consistent with the voltammetric observations, and the systems with low Doxy content showed the most irregular behavior. The release profiles measured by both investigation methods showed a similar trend. In the case of the dissolution profile obtained from the UV-VIS spectrophotometric measurements, a shifting of the equilibrium state was observed, especially in the case of the PLA/Hap_Doxy_3, PLA_Doxy_7, and PLA/Hap_Doxy_7 samples. A possible explanation for this behavior is the continuous addition of PBS during sampling. This dilution of the release medium affected the concentration gradient and thus the Doxy release profiles. To eliminate the shifted equilibrium state, in situ monitoring using DPV proved to be more favorable, as this technique does not disturb the dissolution process in the systems.

Drug release from PLA electrospun mats measured using the voltammetric method yielded a slightly lower release percentage compared to UV-VIS. The different sensitivities of the two methods may explain the difference in the results [31]. The presence of Hap in the nanofiber matrix resulted in a more complex release. Therefore, employing multiple investigation methods is justified for systems of this type. Nevertheless, the advantages of the voltammetric method led to the conclusion that this technique is suitable for investigating the in situ release of Doxy from both PLA and PLA/Hap nanofibers, without the need for sampling and diluting the medium. The short measurement time is another advantage of this method.

#### 3.4.1. Model-Independent Approach

To compare the release profiles obtained using the DPV electrochemical method and the UV-VIS spectrophotometric method, the difference factor (*f*_1_) and similarity factor (*f*_2_) were calculated. The obtained results are shown in Table 1. The similarity factor fulfilled the *f*_2_ > 50 criterion in all cases, which suggests that the DPV method is suitable for real-time monitoring of Doxy release from PLA-based nanofibrous systems. The obtained *f*_1_ values were generally higher than the permissible values, likely due to the variations in the measured amounts of Doxy between the two methods. The values obtained for the PLA/Hap_Doxy_12 sample were the best. The reproducibility of this sample was also high, and the dissolution measured by the spectrophotometric method showed a reasonable fit to the dissolution data series measured by DPV.

#### 3.4.2. Model-Dependent Approach

The release mechanism of Doxy from PLA and PLA/Hap nanofibers was studied using model-dependent models, including zero-order, first-order, Higuchi, Hixson–Crowell, and Korsmeyer–Peppas models (see Table 2 and Table S1). The analysis focused on the first 6 h of the dissolution profiles obtained using the voltammetric method. The model with the best fit was selected based on the highest determination coefficient (R^2^) value. Among the investigated models, the Korsmeyer–Peppas model showed the best fit for the dissolution profiles (Table 2). The mathematical expression for the Korsmeyer–Peppas model is given in Equation (3):

(3) $$\frac{Q_{t}}{Q_{\infty }}=K_{KP}\times t^{n}$$

where *Q_t_* is the amount of drug released at time *t*, *Q_∞_* is the amount of drug released at time ∞, *K_KP_* is the Korsmeyer–Peppas rate constant, and *n* is the diffusional exponent.

According to this model, *n* indicates the mechanism of drug release. If *n* is greater than 0.45, Fickian diffusion is implied. If 0.45 < *n* < 0.89, non-Fickian diffusion takes place, and if *n* ≥ 0.89, it indicates super case II transport [59]. As can be seen in Table 2, *n* < 0.5 for all investigated systems, indicating a Fickian diffusion-controlled mechanism for drug release. The Korsmeyer–Peppas model describes the estimation of drug-release behavior specifically from polymer-based systems [60]. However, the low amount of released Doxy suggests that PLA degradation was insignificant during the release study from PLA-based nanofibers. This allows us to conclude that, in all systems, the diffusion of Doxy molecules into the PBS solution was the predominant mechanism.

In order to evaluate if the results obtained by the two investigation methods (DPV, UV) and fitted by the five kinetic models were different, single-factor and two-factor with replication ANOVA tests were performed. These tests were applied to the values of either the kinetic constant or the coefficient of determination (R^2^). The obtained results are summarized in Table S2.

The single-factor ANOVA test applied to the R^2^ values obtained by fitting the data with the zero-order, first-order, Higuchi, and Hixson–Crowell models showed no significant differences between the obtained results (F < F_crit_, *p* > 0.05). However, by introducing into the test the results obtained by the Korsmeyer–Peppas kinetic model, the differences in R^2^ became significant (F > F_crit_, *p* < 0.05). These results corroborate the data presented in Table 2, where it can be seen that the R^2^ values obtained using the Korsmeyer–Peppas kinetic model were greater than those obtained with the other kinetic models. These results confirm that this model best fits the Doxy release from all investigated matrices. As expected, the single-factor ANOVA test applied to the kinetic constants showed significant differences due to variations in the mathematical equations used to describe each model.

The two-factor with replication ANOVA test applied either to the R^2^ or the kinetic constant values were quite similar. There was a significant difference between the five kinetic models applied (F > F_crit_, *p* < 0.05), and the results obtained by the DPV and UV methods of investigation were quite similar (F < F_crit_, *p* > 0.05). Furthermore, there were no interactions between the five kinetic models and the DPV or UV investigation methods used in the study (F < F_crit_, *p* > 0.05). Consequently, the DPV method of investigation for the release of loaded Doxy from different matrices in different quantities can be successfully used as an alternative study method.

## 4. Conclusions

In the present study, we investigated the in situ release of Doxy physically adsorbed on PLA and PLA/Hap nanofibers in PBS using the differential-pulse voltammetry investigation method, which is rarely used for this purpose. The widely used UV-VIS spectrophotometry technique was used as a control method. The sustained release of Doxy from different Doxy-containing systems was proven by both methods.

Regardless of the chemical composition of the nanofibrous carrier and the Doxy content of the examined system, the kinetic study showed that the release of the active substance was controlled by Fick’s diffusion process, best fitted by the Korsmeyer–Peppas model.

The accuracy of the DPV method was discussed using a model-independent approach. In the case of the PLA/Hap_Doxy_12 sample, the values of the difference and similarity factors best met the criteria.

The DPV method is an analytical technique used for quantitative measurements of analytes, which is appropriate for the in situ, real-time investigation of Doxy release. Based on the presented studies, it can be emphasized that the DPV technique is an excellent, effective, and simple alternative for directly evaluating drug-release profiles and characterizing drug delivery systems, especially for electroactive substances.

## Figures and Tables

**Table 1:** Calculated difference factor (*f*_1_) and similarity factor (*f*_2_) of Doxy release profiles using the UV–VIS and DPV methods.

Sample	Difference Factor (*f*_1_)	Similarity Factor (*f*_2_)
PLA_Doxy_3	16.45	80
PLA_Doxy_7	36.56	72.2
PLA_Doxy_12	33.34	82.24
PLA/Hap_Doxy_3	96.56	53.65
PLA/Hap_Doxy_7	38.27	79.71
PLA/Hap_Doxy_12	4.42	95.35

**Table 2:** Pharmaco-kinetic parameters for Doxy release by Korsmeyer–Peppas model obtained by DPV and UV-VIS measurements.

	Korsmeyer–Peppas					
Samples	DPV			UV		
	*K_KP_*	*n*	R^2^	*K_KP_*	*n*	R^2^
PLA_Doxy_3	4.661	0.169	0.935	9.369	0.063	0.694
PLA_Doxy_7	1.564	0.274	0.899	3.025	0.231	0.909
PLA_Doxy_12	1.365	0.214	0.984	1.842	0.24	0.945
PLA/Hap_Doxy_3	5.139	0.243	0.962	2.311	0.265	0.938
PLA/Hap_Doxy_7	1.119	0.399	0.96	1.464	0.401	0.962
PLA/Hap_Doxy_12	1.506	0.395	0.957	1.8146	0.206	0.9

## Data Availability

The data presented in this study are available on request from the corresponding author.

## Supplementary Materials

The following are available online at https://www.mdpi.com/article/10.3390/jfb14060331/s1, Table S1: Pharmaco-kinetic parameters for Doxy release by Zero-order, Higuchi, First-order, and Hixson-Crowell models obtained by DPV and UV measurements; Table S2. The results of the analysis of variance (ANOVA) for the Doxy release investigated by DPV and UV measurements, and fitted by Zero-order, Higuchi, First-order, Hixson-Crowell, and Korsmeyer-Peppas kinetic models.
