# Supplementary material for: Investigation of the Real-Time Release of Doxycycline from PLA-Based Nanofibers
Source: J Funct Biomater. 2023 Jun 20;14(6):331. doi: 10.3390/jfb14060331 (PMC10299625; doi:10.3390/jfb14060331)
Supplement: Supplementary file 1 [file jfb-14-00331-s001.zip › jfb-2464998-supplementary.pdf]

Investigation of the Real-Time Release of Doxycycline from PLA-Based Nanofibers

Noémi-Izabella Farkas [1], Laura Marincaș [2], Lucian Barbu-Tudoran [3,4], Réka Barabás [5,*] and Graziella Liana Turdean [1,*]

1 Department of Chemical Engineering, Faculty of Chemistry and Chemical Engineering, Babeș-Bolyai University, 11 Arany János Street, 400028 Cluj-Napoca, Romania; noemi.farkas@ubbcluj.ro
2 Department of Chemistry, Faculty of Chemistry and Chemical Engineering, Babeș-Bolyai University, 11 Arany János Street, 400028 Cluj-Napoca, Romania; laura.marincas@ubbcluj.ro
3 Department of Molecular Biology and Biotechnology, Faculty of Biology and Geology, Babeș-Bolyai University, 1 Mihail Kogălniceanu Street, 400084 Cluj-Napoca, Romania; lucian.barbu@ubbcluj.ro
4 National Institute for Research and Development of Isotopic and Molecular Technologies, 67-103 Donath Street, 400293 Cluj-Napoca, Romania
5 Department of Chemistry and Chemical Engineering of Hungarian Line of Study, Faculty of Chemistry and Chemical Engineering, Babeș-Bolyai University, 11 Arany János Street, 400028 Cluj-Napoca, Romania
* Correspondence: reka.barabas@ubbcluj.ro (R.B.); graziella.turdean@ubbcluj.ro (G.L.T.); Tel.: +40-264-593833 (R.B.)

Table S1. Pharmaco-kinetic parameters for Doxy release by Zero-order, Higuchi, First-order, and Hixson-Crowell models obtained by DPV and UV measurements.

	**Zero-order**				**Higuchi**			
	**DPV**		**UV**		**DPV**		**UV**	
**Samples**	k_0	R^2	k_0	R^2	k_H	R^2	k_H	k_H
**PLA_Doxy_3**	0.0143	0.876	0.0084	0.919	0.3471	0.92	0.185	0.185
**PLA_Doxy_7**	0.0096	0.615	0.0136	0.666	0.2542	0.791	0.3517	0.3517
**PLA_Doxy_12**	0.0066	0.929	0.0095	0.754	0.1595	0.98	0.2393	0.2393
**PLA/Hap_Doxy_3**	0.0273	0.75	0.0142	0.749	0.696	0.896	0.3618	0.3618
**PLA/Hap_Doxy_7**	0.0193	0.762	0.0175	0.933	0.491	0.906	0.4173	0.4173
**PLA/Hap_Doxy_12**	0.0119	0.806	0.0066	0.644	0.297	0.928	0.1715	0.1715
	**First-order**				**Hixson-Crowell**			
	**DPV**		**UV**		**DPV**		**UV**	
	k_I	R^2	k_I	R^2	k_{HC}	R^2	k_{HC}	R^2
**PLA_Doxy_3**	-7×10^{-5}	0.881	-4×10^{-5}	0.919	2×10^{-4}	0.879	0.0001	0.919
**PLA_Doxy_7**	-4×10^{-5}	0.618	-6×10^{-5}	0.6727	2×10^{-4}	0.617	0.0002	0.67
**PLA_Doxy_12**	-3×10^{-5}	0.93	-4×10^{-5}	0.759	1×10^{-4}	0.931	0.0002	0.757
**PLA/Hap_Doxy_3**	-1×10^{-4}	0.761	-7×10^{-5}	0.758	5×10^{-4}	0.758	0.0002	0.755
**PLA/Hap_Doxy_7**	-9×10^{-5}	0.769	-8×10^{-5}	0.937	3×10^{-4}	0.766	0.0003	0.936
**PLA/Hap_Doxy_12**	-5×10^{-5}	0.762	-3×10^{-5}	0.646	2×10^{-4}	0.808	0.0001	0.646

Table S2. The results of the analysis of variance (ANOVA) for the Doxy release investigated by DPV and UV measurements, and fitted by Zero-order, Higuchi, First-order, Hixson-Crowell, and Korsmeyer-Peppas kinetic models.

Source of Variation	SS	df	MS	F	P-value	F_{crit}
**Anova: Single Factor in terms of R^2**						
Kinetic models 1-4	0.096783	7	0.013826	1.248613	0.300095	2.249024
Kinetic models 1-5	0.223502	9	0.024834	2.500466	0.019061	2.073351
**Anova: Two-Factor With Replication in terms of R^2**						
Kinetic models 1-5	0.20871512	4	0.052179	5.253823	0.001302	2.557179
Investigation method DPV and UV	0.00903563	1	0.009036	0.909787	0.34476	4.03431
Interactions between kinetic models and investigation methods	0.0057515	4	0.001438	0.144778	0.964478	2.557179
**Anova: Single Factor in terms of kinetic constant, k**						
Kinetic models 1-5	80.38197	9	8.93133	7.145079	1.42E-06	2.073351
**Anova: Two-Factor With Replication in terms of kinetic constant, k**						
Kinetic models 1-5	78.6933	4	19.67332	15.73869	2.12E-08	2.557179
Investigation method DPV and UV	0.257921	1	0.257921	0.206337	0.651619	4.03431
Interactions between kinetic models and investigation methods	1.430755	4	0.357689	0.286152	0.885601	2.557179
